# Supplementary material for: A comparison of dominance rank metrics reveals multiple competitive landscapes in an animal society
Source: Proc Biol Sci. 2020 Sep 9;287(1934):20201013. doi: 10.1098/rspb.2020.1013 (PMC7542799; doi:10.1098/rspb.2020.1013)
Supplement: Supplementary Data, Methods, Figures & Tables (S1-S6) [file rspb20201013supp1.docx]

**A comparison of dominance rank metrics reveals multiple competitive landscapes in an animal society**

Emily J Levy* and Matthew N Zipple*, Emily McLean, Fernando A Campos, Mauna Dasari, Arielle S Fogel, Mathias Franz, Laurence R Gesquiere, Jacob B Gordon, Laura Grieneisen, Bobby Habig, David J Jansen, Niki H Learn, Chelsea J Weibel, Jeanne Altmann, Susan C Alberts^, Elizabeth A Archie^^c^

** First authors contributed equally*

^ *Last authors contributed equally*

*^c^ Corresponding author*

**Supplementary materials**

Table of Contents

[**Dataset used for analysis** 2](#_Toc44407945)

[Table S1 2](#_Toc44407946)

[**Identifying changes in the relationship between simple ordinal and proportional ranks over time** 5](#_Toc44407947)

[Methods 5](#_Toc44407948)

[Table S2 5](#_Toc44407949)

[Results 6](#_Toc44407950)

[Figure S3 7](#_Toc44407951)

[**Other supplementary figures and tables** 8](#_Toc44407952)

[Figure S4 8](#_Toc44407953)

[Table S5 9](#_Toc44407954)

[Table S6 10](#_Toc44407955)

[**References** 11](#_Toc44407956)

# **Dataset used for analysis**

**Table S1**. Trait descriptions, ΔAICs, and study information for the 20 traits re-analyzed in the present study.

| Trait^a^ | Originally Identified Rank Effect^b^ | Study Duration (years) | # of Social Groups | AIC Null Model | AIC Simple Ordinal Model | AIC Proportional Model | Preferred Model^c^ | \|∆AIC\| Simple Ordinal vs. Proportional^d^ | \|∆AIC\| Preferred vs. Null^d^ | Ref^ |
| --- | --- | --- | --- | --- | --- | --- | --- | --- | --- | --- |
| *Adult male traits* | | | | | | | | | | |
| Percent of consortships obtained | Higher-ranking males obtain more consortships | 12 | 7 | -21610 | -21768 | -21741 | Simple Ordinal | 27 | 158 | [1] |
| Fecal testosterone | Higher-ranking males have higher testosterone levels | 9 | 5 | -416 | -443.1 | -418.2 | Simple Ordinal | 24.9 | 27.1 | [2]^ |
| Wound healing | Higher-ranking males have faster rates of wound healing | 28 | 11 | 3646 | 3630 | 3638 | Simple Ordinal | 8 | 16 | [3] |
| Monthly injury risk | Injury incidence is related to a quadratic rank term, with males ranked 3-6 having the highest rates of injury | 28 | 11 | 3539.6 | 3534.2 | 3540.9 | Simple Ordinal | 6.7 | 5.4 | [3] |
| Age at testicular enlargement | Males with higher-ranking mothers achieve testicular enlargement at younger ages | 22 | 9 | 1044.4 | 1042.1 | 1045.2 | Simple Ordinal | 3.1 | 2.3 | [4] |
| Fecal glucocorticoid levels | Higher-ranking males have lower glucocorticoid levels (except for the alpha male) | 9 | 5 | -1223.4 | -1227 | -1223.2 | Simple Ordinal | 3.8 | 3.6 | [2]^ |
| *Immature male traits* | | | | | | | | | | |
| Fecal glucocorticoid levels | Subadult sons of higher-ranking mothers have lower glucocorticoid levels | 4 | 5 | 141.8 | 137.4 | 137.6 | Neither | 0.2 | 4.4 | [5] |
| Body size | Juvenile males with higher-ranking mothers have larger body size for their age | 1 | 2 | -53.5 | -69.4 | -62.2 | Simple Ordinal | 7.2 | 15.9 | [6] |
| *Adult female traits* | | | | | | | | | | |
| Post-partum amenorrhea duration | Higher-ranking females have shorter periods of post-partum amenorrhea | 36 | 13 | 5559.8 | 5542.4 | 5533.9 | Proportional | 8.5 | 25.9 | [7]^ |
| Inter-birth interval duration | Higher-ranking females have shorter inter-birth intervals | 36 | 13 | 5912.6 | 5899.1 | 5897.8 | Neither | 1.3 | 14.8 | [7]^ |
| Monthly injury risk | Higher-ranking (proportional) females have a lower risk of injury | 29 | 12 | 4746 | 4742.7 | 4735.7 | Proportional | 7.1 | 10.3 | [8]^ |
| Prenatal fecal estrogen levels | Higher-ranking females have higher prenatal estrogen levels | 1.4 | 5 | -36.4 | -42.2 | -38 | Simple Ordinal | 4.2 | 5.8 | [9] |
| Fecal glucocorticoid levels | Higher-ranking females (proportional) have lower glucocorticoid levels | 17 | 12 | -9727.4 | -9726.1 | -9728.8 | Proportional | 3.0 | 1.4 | [10]^ |
| Age at menarche | Females with higher-ranking mothers achieve menarche at younger ages | 26 | 9 | 129.2 | 126.6 | 127.3 | Neither | 0.7 | 2.6 | [4] |
| Relative infant survival | Higher-ranking females have higher rates of infant survival | 16 | 6 | 39.9 | 35.9 | 37.1 | Neither | 1.2 | 4.0 | [11] |
| Sexual swelling length | Higher-ranking females have longer sexual swellings | 1.5 | 5 | 699.5 | 697.2 | 697.1 | Neither | 0.1 | 2.4 | [12]^ |
| Social connectedness to males | Higher-ranking females are more socially connected to males | 16 | 8 | 4131.6 | 4098.5 | 4101.9 | Simple Ordinal | 3.3 | 33.1 | [13]^ |
| Frequency of received grooming | Higher-ranking females receive more grooming | 2 | 5 | 494.1 | 476.0 | 478.4 | Simple Ordinal | 2.3 | 18.1 | [14]^ |
| *Immature female traits* | | | | | | | | | | |
| Initiation rate by infants | No statistically significant effect of maternal rank on infant initiation rate | 1.4 | 5 | 216.6 | 217.5 | 214.1 | Proportional | 3.4 | 2.5 | [9] |
| *Infant male and female traits* | | | | | | | | | | |
| Time off nipple | Infants of higher-ranking females tended to spend more time on the nipple | 1.4 | 5 | -65.1 | -71.9 | -68.7 | Simple Ordinal | 3.2 | 6.8 | [9] |

^a^ For traits measured in immature individuals, rank was measured as maternal rank

^b^ Simple ordinal rank unless otherwise noted

^c^ “Neither” if |∆AIC_simple ordinal-proportional_| <2

^d^  These columns were calculated prior to rounding the model AIC values

^ Indicates that dataset is publicly available on Dryad

# **Identifying changes in the relationship between simple ordinal and proportional ranks over time**

**Methods**: In long-term studies, hierarchy size varies over time and across social groups. This variation should simultaneously weaken the relationship between simple ordinal and proportional rank and increase our ability to measure different competitive processes in social groups. To test the prediction that the relationship between ordinal and proportional ranks weakens as studies progress, we measured the correlation between monthly simple ordinal and proportional ranks in the Amboseli Baboon Research Project dataset over increasingly longer periods of time.

Specifically, for each social group we have studied (N = 17 groups), we calculated the R^2^ values from linear models that predicted proportional rank as a function of simple ordinal rank using increasingly larger datasets. The decision of which metric to use as the predictor variable, in this case simple ordinal rank, and which as the response variable, in this case proportional rank, was random and had no effect on the results of these analyses. We began by calculating this correlation using only rank data from the first month that a group was under observation (R^2^ necessarily = 1). We then repeated this R^2^ calculation iteratively, each time drawing on ever-larger datasets, by adding data in 12-month increments (i.e. 13 total months, 25 total months, 37 total months, etc.), until we reached the last available dataset of ranks for a group (see Table S2 an example dataset). This method allowed us to track the strength of the predictive relationship between ordinal and proportional ranks as the study progressed.

These analyses included a total of 17 social groups that have been studied over the last 40+ years (thin black lines in Figure S3). We also repeated the same approach, combining data from all social groups into a single analysis (thick grey lines in Figure S3), allowing us to qualitatively compare patterns of change in the relationship between ordinal and proportional ranks both within social groups and across the study population. Note that at the start of the project, only a single social group was followed (Alto’s group). As a result, the grey line starts at an R^2^ value of 1. If multiple study groups with different group sizes had been followed at the beginning of the study, the R^2^ value at the beginning of the project would have been < 1.

**Table S2**. Example dominance ranks from seven individuals across three months and how these data would be used to calculate R^2^ values via a linear model for predicting proportional rank from ordinal rank. To calculate the relationship between simple ordinal and proportional ranks across the 3 months in the table (January, February, and March 2016), every row in this dataset would be used in a linear model in which proportional rank is the response variable and simple ordinal rank is the predictor variable. Individual identity did not factor into the model or calculation of R^2^, so the switch in rank order between individuals C, D, and E from February to March 2016 is irrelevant. What does, however, reduce R^2^ is the addition of individual G to the group in February 2016, and the loss of individual G from the group in March 2016.

| **Individual Identity** | **Year-Month** | **Simple Ordinal Rank** | **Proportional Rank** |
| --- | --- | --- | --- |
| A | Jan-2016 | 1 | 1 |
| B | Jan-2016 | 2 | 0.8 |
| C | Jan-2016 | 3 | 0.6 |
| D | Jan-2016 | 4 | 0.4 |
| E | Jan-2016 | 5 | 0.2 |
| F | Jan-2016 | 6 | 0 |
| A | Feb-2016 | 1 | 1 |
| B | Feb-2016 | 2 | 0.8333 |
| D | Feb-2016 | 3 | 0.6667 |
| C | Feb-2016 | 4 | 0.5 |
| E | Feb-2016 | 5 | 0.3333 |
| F | Feb-2016 | 6 | 0.1667 |
| G | Feb-2016 | 7 | 0 |
| A | Mar-2016 | 1 | 1 |
| B | Mar-2016 | 2 | 0.8 |
| D | Mar-2016 | 3 | 0.6 |
| C | Mar-2016 | 4 | 0.4 |
| E | Mar-2016 | 5 | 0.2 |
| F | Mar-2016 | 6 | 0 |

**Results**: As predicted, the correlation between simple ordinal and proportional ranks both within and across study groups decreased as the length of study increased (Figure S3). This is because the size of adult female and male hierarchies changed over time. Variation in hierarchy size, in turn, decouples our density-independent rank metric (proportional) from our density-dependent rank metric (simple ordinal). This decline in R^2^ as the length of study increased was seen in each group individually and across all study groups when all data were combined (i.e., across the study population).

The decline in R^2^ over time was apparent in both male and female ranks, although the decline occurred more quickly and less linearly in the male rank data than in the female rank data. This sex difference, which we did not predict, prompted us to form two post-hoc predictions to explain it. (1) Baboon groups contained fewer adult males than females; hence hierarchy sizes are smaller for males than for females. The addition of one individual to a small hierarchy changes all members’ proportional ranks more than the addition of one individual to a large hierarchy (Figure S4). Thus, if we assume that different-sized groups have comparable rates of maturation, death, and dispersal, the relationship between simple ordinal and proportional ranks would be weaker in smaller hierarchies than larger hierarchies. (2) Changes in male hierarchy size were more common than changes in female hierarchy size due to frequent male dispersal, and all changes in hierarchy size reduce the relationship between simple ordinal and proportional ranks. Together, we would expect these two sex differences – in average hierarchy size and in the frequency of changes in hierarchy size – to lead to differences in the relationship between simple ordinal and proportional rank between males and females (Figure S4).

We performed post-hoc analyses and confirmed both of our predictions. Of the 1,637 group-months for which we had rank data for both males and females, adult males outnumbered adult females in only 14 months (<1% of group-months; mean # of females - mean # of males ± SD =7.4 ± 0.1, p<0.0001 in one-sample, two-tailed t-test). Additionally, on average, the number of adult males in a social group changed more from one month to the next as compared to the number of adult females (mean absolute change in # adult males per month ± SD = 0.59 ± 0.02, mean in adult females ± SD = 0.25 ± 0.01 , p<0.0001 in two-sample, two-tailed t-test).

**
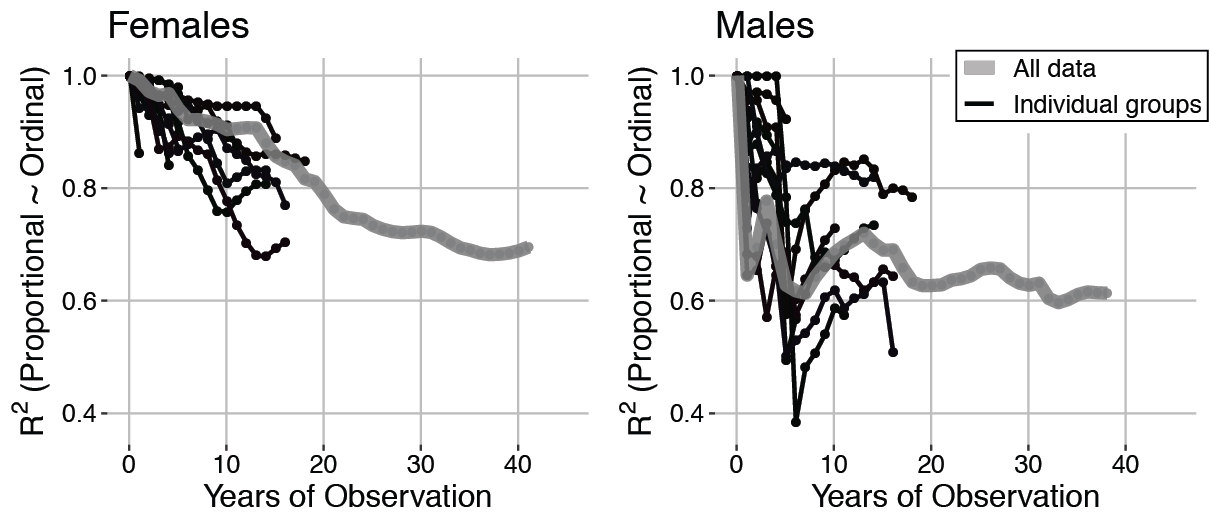
**

**Figure S3**. The relationship between simple ordinal rank (referred to in the figure as ‘ordinal rank')and proportional ranks weakens as the period of observation increases in both females and males. Black points and lines indicate changes in R^2^ for every social group over time, and the grey points and lines indicate changes across all social groups (i.e. across the study population) using pooled data from all individuals in all social groups. At each point, R^2^ was calculated from the cumulative dataset (e.g. the grey point at 13 months includes data from all 13 months across all individuals in all social groups). The grey line extends farther than any black line because the black lines represent individual social groups, which are not permanent due to fissions and fusions, whereas the grey line represents the full dataset.

# **Other supplementary figures and tables**

**Figure S4**. The relationship between simple ordinal rank (referred to in the figure as ‘ordinal rank’) and proportional ranks is weakened when hierarchy size changes. We simulated groups that included varying numbers of adults (range 2-20) and assigned simple ordinal and proportional ranks to each individual for one rank period (one month). We then added varying numbers of individuals to the group, and again assigned simple ordinal and proportional ranks to each individual for a second ranking period. We then calculated the R^2^ value from a model that predicted proportional rank as a function of simple ordinal rank, including all ranks from both ranking periods. The relationship between simple ordinal and proportional ranks is less robust to greater changes in hierarchy size and less robust to changes in smaller starting hierarchies. The situation described in the introduction, in which four males join an existing group of five males, is marked with an asterisk.

**Table S5**. Theoretical distribution of resources under density-independent and density-dependent competition for two group sizes to demonstrate theoretical differences between ordinal and proportional ranks.

| **Density-Independent Competition**:  Competition for Food | | | | **Density-Dependent Competition**:  Competition for Mates | | | |
| --- | --- | --- | --- | --- | --- | --- | --- |
| Group Size = 5 | | Group Size = 9 | | Group Size = 5 | | Group Size = 9 | |
| Ord. Rank  [Prop. Rank] | Food Obtained | Ord Rank [Prop Rank] | Food Obtained | Ord. Rank  [Prop. Rank] | Mates Obtained | Ord. Rank [Prop Rank] | Mates Obtained |
| 1  [1.00] | 3 | 1  [1.00] | 3 | 1  [1.00] | 1 | 1  [1.00] | 1 |
|  | | 2  [0.88] | 2.75 |  | | 2  [0.88] | 1 |
| 2  [0.75] | 2.5 | 3  [0.75] | 2.5 | 2  [0.75] | 1 | 3  [0.75] | 1 |
|  | | 4  [0.63] | 2.25 |  | | 4  [0.63] | 0 |
| **3^*^**  **[0.50]** | **2.0** | **5**  **[0.50]** | **2.0** | **3^*^**  **[0.50]** | **1** | **5**  **[0.50]** | **0** |
|  | | 6  [0.38] | 1.75 |  | | 6  [0.38] | 0 |
| 4  [0.25] | 1.5 | 7  [0.25] | 1.5 | 4  [0.25] | 0 | 7  [0.25] | 0 |
|  | | 8  [0.13] | 1.25 |  | | 8  [0.13] | 0 |
| 5  [0.00] | 1.0 | 9  [0.00] | 1.0 | 5  [0.00] | 0 | 9  [0.00] | 0 |
| Per Capita = 2^^^ | | Per Capita = 2 | | Per Capita = 0.6^^^ | | Per Capita = 0.333 | |

* The middle-ranking individual in each group is bolded for comparison. Under density-independent competition, access to resources is determined by proportional rank. The middle-ranking animal obtains 2 units of food regardless of ordinal rank or hierarchy size. Under density-dependent selection, access to resources is determined by simple ordinal rank. The middle-ranking animal obtains 1 mate when its simple ordinal rank is 3 but 0 mates when its ordinal rank is 5.

^ Under density-independent competition, per capita resource access remains constant as hierarchy size increases. Under density-dependent competition, per capita resource access declines as hierarchy size increases.

**Table S6**. Trait descriptions and model equations for the 20 traits re-analyzed in the present study.

| Trait^*^ | Model used in analysis |
| --- | --- |
| Percent of consortships obtained (M) | Percent consortships obtained ~ Rank |
| Fecal testosterone (M) | Log(Testosterone) ~ Rank + Alpha Status + Hierarchy Stability + Season + Temperature + Age + (1\|ID) |
| Wound healing (M) | Wound Healing Time ~ Rank + Type of Wound + Adult Group Size + Change in Data Recording |
| Monthly injury risk (M) | Number of Injuries ~ Rank^2^ + (1\|ID) |
| Age at testicular enlargement (M) | Age at Testicular Enlargement ~ Hybrid Score + # of Excess Females + Maternal Rank + (1\|Group ID) |
| Fecal glucocorticoid levels (M) | Log(Fecal Glucocorticoid) ~ Rank + Alpha Status + Hierarchy Stability + Age + Season + Temperature + (1\|ID) |
| Fecal glucocorticoid levels (IM) | Fecal Glucocorticoid ~ Maternal Rank + Age + Parity + Immature’s Simple Ordinal Rank |
| Body size (IM) | Body Size-for-Age (calculated using Lowess) ~ Rank |
| Post-partum amenorrhea duration (F) | PPA Duration ~ Primiparous/Multiparous + Maternal Age + Group Size + Rain Fall + Habitat + Sex of Offspring + (1\| Maternal ID) + (1\|Group ID) + (1\|Year) |
| Inter-birth interval duration (F) | IBI Duration ~ Primiparous/Multiparous + Maternal Age + Group Size + Rain Fall + Habitat + Sex of Offspring + (1\| Maternal ID) + (1\|Group ID) + (1\|Year) |
| Monthly injury risk (F) | Injured or Not ~ Rank + Age + Reproductive State + Group Fission Status + (1\|ID) + (1\|Year) |
| Prenatal fecal estrogen levels (F) | Fecal Estrogen ~ Rank |
| Fecal glucocorticoid levels (F) | Log(Fecal Glucocorticoid) ~ Rank + Age + Season + Reproductive State + Adult Female Group Size + Adult Female Group Size^2^ + Storage Time as Fecal Powder + Storage Time in Methanol + (1\|ID) + (1\|Group ID) + (1\|Hydroyear) |
| Age at menarche (F) | Age at Menarchy ~ # of Mature Maternal Sisters + Hybrid Score + # of Mature Females + (1\|Group ID) |
| Relative infant survival (F) | Relative Infant Survival ~ Maternal Rank |
| Sexual swelling length (F) | Sexual Swelling Length ~ Cycles Since Resumption + Days Since Last Major Rain + Cycles Since Resumption*Days Since Last Major Rank + Rank + (1\|ID) |
| Social connectedness to males (F) | Social Connectedness to Males ~ Age + Group Size + Maternal Presence + # of Maternal Sisters + # of Daughters + Social Connectedness to Females + Rank + (1\|ID) |
| Frequency of received grooming (F) | Frequency of Grooming ~ Age + Rank + (1\|Group ID) |
| Initiation rate by infants (IF) | Initiation Rate ~ Infant Sex + Maternal Parity + Maternal Rank |
| Time off nipple (IB) | Prop Time Off Nipple ~ Maternal Rank |

^*^ M = trait measured in adult males; F = trait measured in adult females; B = trait measured in both males and females, no differentiation by sex; I = trait measured in immature individuals, rank measured as maternal rank

**References:**

1. Alberts SC, Buchan JC, Altmann J. 2006 Sexual selection in wild baboons: from mating opportunities to paternity success. *Anim. Behav.* **72**, 1177–1196. (doi:10.1016/j.anbehav.2006.05.001)

2. Gesquiere LR, Learn NH, Simao MCM, Onyango PO, Alberts SC, Altmann J. 2011 Life at the top: rank and stress in wild male baboons. *Science* **333**, 357–60. (doi:10.1126/science.1207120)

3. Archie EA, Altmann J, Alberts SC. 2012 Social status predicts wound healing in wild baboons. *Proc. Natl. Acad. Sci. U. S. A.* **109**, 9017–22. (doi:10.1073/pnas.1206391109)

4. Charpentier MJE, Tung J, Altmann J, Alberts SC. 2008 Age at maturity in wild baboons: genetic, environmental and demographic influences. *Mol. Ecol.* **17**, 2026–2040. (doi:10.1111/j.1365-294X.2008.03724.x)

5. Onyango PO, Gesquiere LR, Wango EO, Alberts SC, Altmann J. 2008 Persistence of maternal effects in baboons: Mother’s dominance rank at son’s conception predicts stress hormone levels in subadult males. *Horm. Behav.* **54**, 319–324. (doi:10.1016/j.yhbeh.2008.03.002)

6. Altmann J, Alberts SC. 2005 Growth rates in a wild primate population: ecological influences and maternal effects. *Behav. Ecol. Sociobiol.* **57**, 490–501. (doi:10.1007/s00265-004-0870-x)

7. Gesquiere LR, Altmann J, Archie EA, Alberts SC. 2018 Interbirth intervals in wild baboons: environmental predictors and hormonal correlates. *Am. J. Phys. Anthropol.* **166**, 107–126. (doi:10.1002/ajpa.23407)

8. Archie EA, Altmann J, Alberts SC. 2014 Costs of reproduction in a long-lived female primate: Injury risk and wound healing. *Behav. Ecol. Sociobiol.* **68**, 1183–1193. (doi:10.1007/s00265-014-1729-4)

9. Nguyen N, Gesquiere L, Alberts SC, Altmann J. 2012 Sex differences in the mother-neonate relationship in wild baboons: Social, experiential and hormonal correlates. *Anim. Behav.* **83**, 891–903. (doi:10.1016/j.anbehav.2012.01.003)

10. Levy E *et al.* In press. Higher dominance rank is associated with lower glucocorticoids in wild female baboons: a rank metric comparison. *Horm. Behav.*

11. Silk JB, Alberts SC, Altmann Je. 2003 Social bonds of female baboons enhance infant survival. *Science (80-. ).* **302**, 1231–1234. (doi:10.1126/science.1088474)

12. Fitzpatrick CL, Altmann J, Alberts SC. 2014 Sources of variance in a female fertility signal: Exaggerated estrous swellings in a natural population of baboons. *Behav. Ecol. Sociobiol.* **68**, 1109–1122. (doi:10.1007/s00265-014-1722-y)

13. Archie EA, Tung J, Clark M, Altmann J, Alberts SC. 2014 Social affiliation matters: both same-sex and opposite-sex relationships predict survival in wild female baboons. *Proc R. Soc L. B Biol. Sci* **281**, 20141261. (doi:10.1098/rspb.2014.1261)

14. Akinyi MY, Tung J, Jeneby M, Patel NB, Altmann J, Alberts SC. 2013 Role of grooming in reducing tick load in wild baboons (Papio cynocephalus). *Anim. Behav.* **85**, 559–568. (doi:10.1016/j.anbehav.2012.12.012)
